# P-559. Characteristics of Women Initiating CAB+RPV LA in the OPERA Cohort

**DOI:** 10.1093/ofid/ofae631.758

**Published:** 2025-01-29

**Authors:** Jessica A Altamirano, Brooke Levis, Cindy Markarian, Quateka Cochran, Courtney Sherman, Mona-Gekanju Toeque, Laura Armas, Gayathri Sridhar, Vani Vannappagari, Kimberley Brown, Jennifer S Fusco

**Affiliations:** CAN Community Health, Miami, Florida; Epividian, Inc, Montreal, Quebec, Canada; AIDS Healthcare Foundation, Long Beach, California; Aids Healthcare Foundation, Miami Beach, Florida; CAN Community Health, Miami, Florida; AIDS Health Foundation, Los Angeles, California; Human Centered Consulting & Care, McKinney, Texas; ViiV Healthcare, Fairfax, Virginia; ViiV Healthcare, Fairfax, Virginia; ViiV Healthcare, Fairfax, Virginia; Epividian, Inc., Durham, North Carolina

## Abstract

**Background:**

Cabotegravir + rilpivirine long-acting (CAB+RPV LA) injectable administered once a month or every two months is the first complete LA antiretroviral (ART) regimen approved for HIV-1 treatment. It is indicated for treatment experienced individuals with viral loads (VL) < 50 copies/mL. CAB+RPV LA has attributes that may make it a good option for women, who represent ∼20% of people with HIV in the US and may experience unique challenges with HIV treatment. CAB+RPV LA may reduce psychosocial concerns around adherence, stigma, and disclosure associated with daily oral therapy. We assessed baseline characteristics and dosing schedules among women receiving CAB+RPV LA in the OPERA^®^ cohort.

**Figure:**
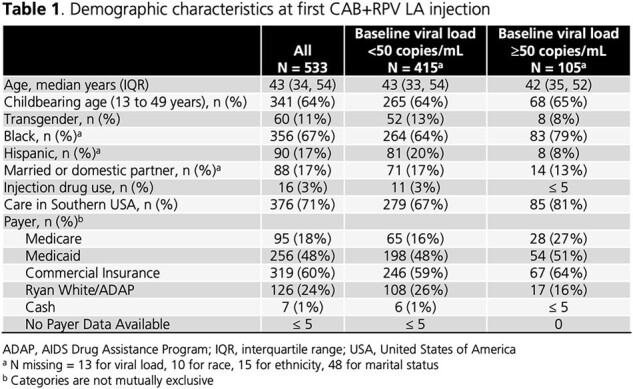

**Methods:**

Adult and adolescent women ≥ 13 years old with HIV who received ≥ 1 CAB+RPV LA injection between 21JAN2021-31AUG2023 were included. Baseline characteristics and dosing schedules were described overall and stratified by VL at first injection (< 50 vs. ≥ 50 copies/mL).

**Figure:**
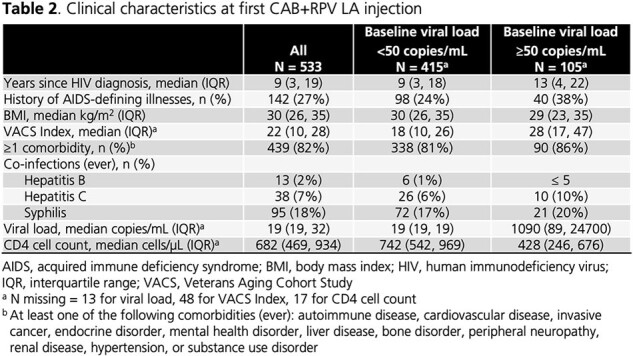

**Results:**

Of 533 women in 19 states and Puerto Rico starting CAB+RPV LA (78% with VL < 50 copies/mL, 20% with VL ≥ 50 copies/mL, 2% missing VL), 11% were transgender, 67% were Black, 17% were Hispanic, and 64% were of childbearing age (13-49 years; **Table 1**). Almost all had medical coverage (1% paid cash). Median years since HIV diagnosis was 9 (IQR: 3, 19), median BMI was 30 (IQR: 26, 35), 82% had ≥ 1 comorbidity, and 27% had an AIDS-defining illness (**Table 2**). Women received a median of 2 (IQR: 1, 2) core agents prior to CAB+RPV LA and 67% were on INSTIs immediately prior to CAB+RPV LA (**Table 3**). Of 508 women with dosing information, 96% were on Q2M schedules. Compared to women with VL < 50 copies/mL, women with VL ≥ 50 copies/mL had longer disease duration, higher VACS scores, and lower CD4 cell counts. They were more likely to have an AIDS-defining illness and have experienced ≥ 3 core agents.

**Figure:**
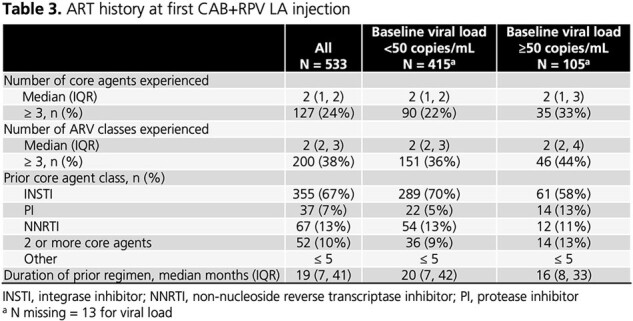

**Conclusion:**

To date, > 500 women in OPERA have received CAB+RPV LA injections; most started the regimen early in their HIV treatment journey, with notable diversity across racial and ethnic groups. One in five women started the regimen while having a detectable VL; these women had more advanced HIV and were more treatment-experienced, with potentially fewer ARV options. Our findings provide unique insights about this important but understudied subpopulation of people with HIV.

**Disclosures:**

**Jessica A. Altamirano, MD, FIDSA**, Gilead Sciences: Speaker's Bureau **Brooke Levis, PhD**, EMD Serono: Research support to my employer|Gilead Sciences: Research support to my employer|Merck & Co.: Research support to my employer|TheraTechnologies: Research support to my employer|ViiV Healthcare: Research support to my employer **Laura Armas, MD, FACP, AAHIVS**, Gilead: Advisor/Consultant|Gilead: Grant/Research Support **Gayathri Sridhar, MBBS, MPH, PhD**, GlaxoSmithKline: Stocks/Bonds (Public Company)|ViiV Healthcare: Full Time Employee **Vani Vannappagari, MBBS, MPH, PhD**, GSK: Stocks/Bonds (Public Company)|ViiV Healthcare: Full time Employee|ViiV Healthcare: Stocks/Bonds (Public Company) **Kimberley Brown, PharmD**, GSK: Stocks/Bonds (Public Company)|ViiV Healthcare: Employee **Jennifer S. Fusco, BS**, EMD Serono: Research support to my employer|Gilead Sciences: Research support to my employer|Merck & Co.: Research support to my employer|TheraTechnologies: Research support to my employer|ViiV Healthcare: Research support to my employer

